# Multidimensional library for the improved identification of per- and polyfluoroalkyl substances (PFAS)

**DOI:** 10.1038/s41597-024-04363-0

**Published:** 2025-01-25

**Authors:** Kara M. Joseph, Anna K. Boatman, James N. Dodds, Kaylie I. Kirkwood-Donelson, Jack P. Ryan, Jian Zhang, Paul A. Thiessen, Evan E. Bolton, Alan Valdiviezo, Yelena Sapozhnikova, Ivan Rusyn, Emma L. Schymanski, Erin S. Baker

**Affiliations:** 1 Department of Chemistry, University of North Carolina at Chapel Hill, Chapel Hill, NC USA; 2 Immunity, Inflammation, and Disease Laboratory, National Institute of Environmental Health Sciences, Durham, NC 27709 USA; 3 National Center for Biotechnology Information, National Library of Medicine, National Institutes of Health, Bethesda, MD 20894 USA; 4 Interdisciplinary Faculty of Toxicology, Texas A&M University, College Station, TX 77843 USA; 5 Department of Veterinary Physiology and Pharmacology, Texas A&M University, College Station, TX 77843 USA; 6 Agricultural Research Service, U.S Department of Agriculture, Wyndmoor, PA 19038 USA; 7 Luxembourg Centre for Systems Biomedicine (LCSB), University of Luxembourg, 6 Avenue du Swing, 4367 Belvaux, Luxembourg

**Keywords:** Environmental monitoring, Environmental impact

## Abstract

As the occurrence of human diseases and conditions increase, questions continue to arise about their linkages to chemical exposure, especially for per-and polyfluoroalkyl substances (PFAS). Currently, many chemicals of concern have limited experimental information available for their use in analytical assessments. Here, we aim to increase this knowledge by providing the scientific community with multidimensional characteristics for 175 PFAS and their resulting 281 ion types. Using a platform coupling reversed-phase liquid chromatography (RPLC), electrospray ionization (ESI) or atmospheric pressure chemical ionization (APCI), drift tube ion mobility spectrometry (IMS), and mass spectrometry (MS), the retention times, collision cross section (CCS) values, and *m/z* ratios were determined for all analytes and assembled into an openly available multidimensional dataset. This information will provide the scientific community with essential characteristics to expand analytical assessments of PFAS and augment machine learning training sets for discovering new PFAS.

## Background & Summary

Per- and polyfluoroalkyl substances (PFAS) are a class of synthetic, fluorinated chemicals used in a variety of consumer products and industrial processes over the last 70 years^1^. While there are many definitions of which structural elements constitute classification as a PFAS, the community largely accepts that proposed by the Organisation for Economic Co-operation and Development (OECD) which includes any chemical having “at least one saturated CF_2_ or CF_3_ moiety”^2,3^. This definition is the most inclusive, encompassing nearly 7 million compounds identified by the National Institutes of Health’s (NIH) open chemical database, PubChem^2,4^. This definition includes traditional legacy PFAS compounds like perfluorooctanoic acid (PFOA) and perfluorosulfonic acid (PFOS), replacement PFAS such as hexafluoropropylene oxide dimer acid (HFPO-DA, or commonly GenX), as well as short chain fluorinated compounds such as trifluoroacetic acid (TFA), and organofluorine pharmaceuticals and pesticides^2,4,5^. PFAS have special chemical and physical properties due to their characteristic carbon-fluorine bonds, making them both water and oil repellent, resistant to thermal or chemical degradation, and highly useful surfactants^5^. As a result, PFAS have been detected in a variety of widely used products, including fast-food packaging, cosmetics, water-resistant clothing, and fire-fighting foams^5–8^. The caveat to their utility is that some PFAS are resistant to degradation in the environment and also bioaccumulate. PFAS have thus been detected in virtually all environmental samples including soil^9^ and water systems, and their prolific abundance in the environment promotes both direct and indirect routes of exposure to humans (*e.g*., drinking water and inhalation of house dust)^10,11^. As a consequence, PFAS are now routinely observed in human serum, breastmilk, and other biological tissues^12–16^. Furthermore, multiple studies reported associations between exposure to PFAS and a variety of adverse health effects, including decreased immune function^17^, lipid dysregulation^18^, pre- and post-natal development issues^19^, and cancer^20^. Therefore, many PFAS are considered to be both persistent, mobile, and toxic (PMT) compounds and very persistent very mobile (vPvM) substances by the European Union^2,21^.

Despite the suspected adverse health implications and prevalence of PFAS in the environment, the United States regulates the concentration of only six PFAS, exclusively in drinking water^22^. To evaluate their presence and concentrations, common targeted analytical methods for PFAS utilize liquid chromatography-mass spectrometry (LC-MS) platforms with a triple quadrupole mass spectrometer, typically covering less than 50 of these analytes^23^. However, as the list of PFAS continues to grow in number and chemical complexity, more comprehensive and robust analytical techniques are becoming essential to evaluate a problem of such scale. The use of non-targeted approaches has therefore increased to detect novel PFAS in products, humans, and the environment^5^. These non-targeted approaches commonly include LC separations coupled with time-of-flight and Orbitrap mass analyzers to provide high resolution mass spectrometry (HRMS) measurements. However, even these measurements have limitations in PFAS identification as their similarity in mass to other molecules in complex mixtures and the existence of isomeric PFAS, or those with the same monoisotopic mass and molecular formula, challenge LC-HRMS measurements. Ion mobility spectrometry (IMS) has therefore been coupled with traditional LC-HRMS methods to aid in these distinctions^8,24–26^. Drift tube IMS (DTIMS) is a rapid gas-phase separation technique in which ions are separated based on their size and shape^27,28^. In DTIMS, ions traverse the length of the drift cell through an inert buffer gas and under the influence of a uniform electric field^27^. The resulting measurement is an ion’s drift time, which can be used to calculate the collision cross section (CCS) value of the ion, providing a measurement of an ion’s gas phase surface area^27^. CCS values are an important metric for confident PFAS identifications as they are highly reproducible across instruments and laboratory conditions (often within <1% error)^27,28^.

This study uses a LC-DTIMS-HRMS platform to contribute to characterizations of 175 PFAS with authentic standards in a multidimensional manner. While previous analytical characteristics for 145 PFAS have been reported in several papers for negative mode ionization, this manuscript combines all those values and adds information for 30 new commercial PFAS standards and other ion types (e.g., [M-H]^−^, [M-H-CO_2_]^−^, [M + Cl]^−^, [M + H]^+^, [M + NH_4_]^+^, [M + Na]^+^, [M + K]^+^, [M-H-CO_2_-HF]^−^, [M-C_2_H_4_-OH]^−^, [M-CH_2_-CO_2_]^−^, [M-2H + Na]^−^, [M-2H + K]^−^, and [M + CH_3_O]^−^) observed for the new and previously analyzed PFAS^24,29,30^. Specifically, the multidimensional dataset includes 281 PFAS ions types and related multimers observed in negative and positive modes, as well as those formed by both electrospray ionization (ESI) and atmospheric pressure chemical ionization (APCI) sources.

## Methods

### Standards and reagents

**Fig. 1:**
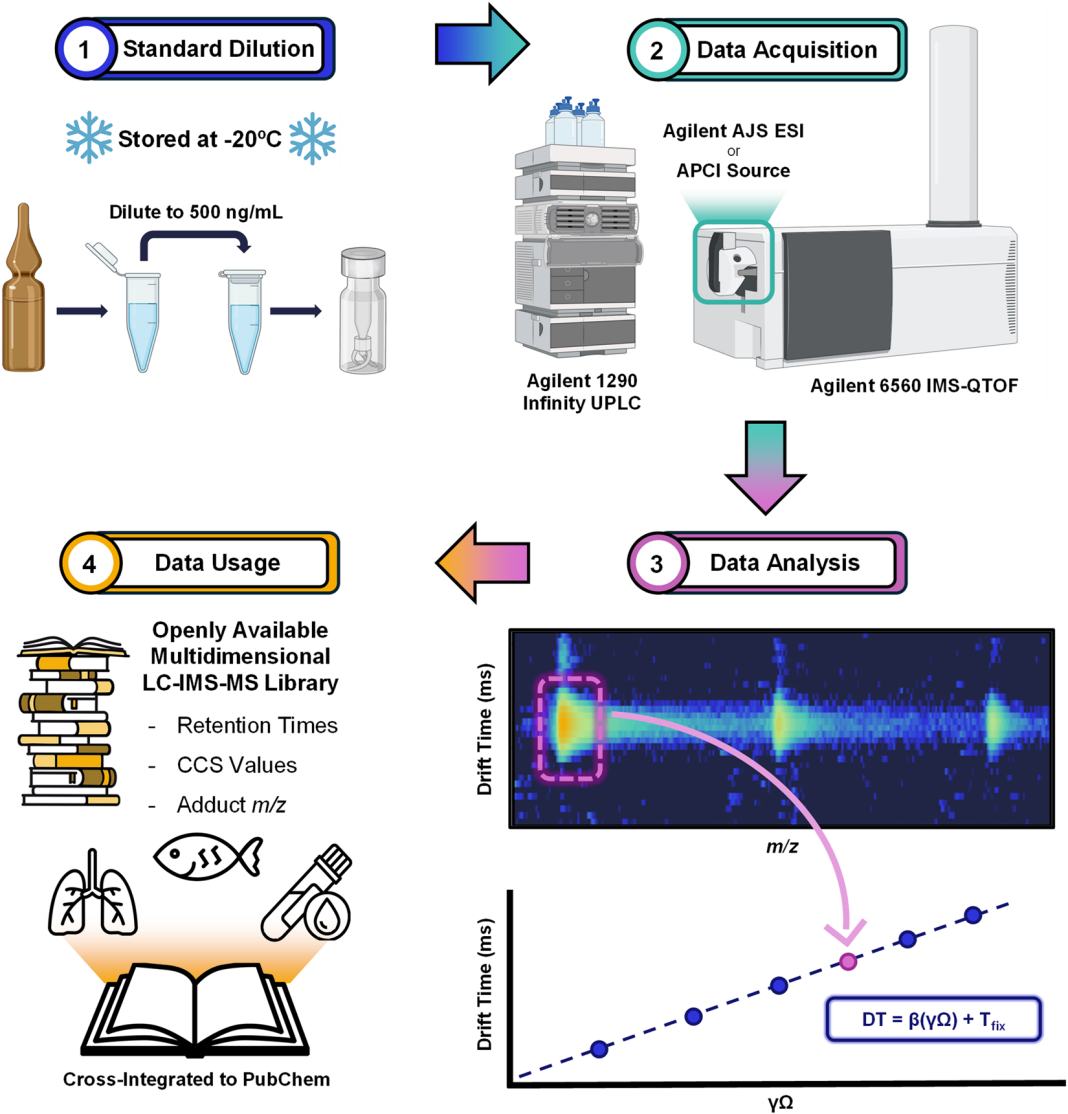
Experimental workflow for RPLC-IMS-HRMS library construction. 1 – Standard Dilution: Commercially purchased PFAS standards were diluted 100-fold and transferred to LC vials. 2 – Data Acquisition: Standards were run via either flow injection or a chromatographic gradient, ionized via ESI or APCI and subsequent data acquired on an Agilent 6560 IMS-QTOF. 3 – Data Analysis: Data was analyzed using Agilent IMS-Browser Version 10.00 and integration of the nested spectra results in *m/z* and drift times (converted to CCS value via the Mason-Schamp equation) for each observed ion. 4 – Data Usage: Open-access multidimensional RPLC-IMS-HRMS data can be used for targeted analysis of PFAS in a variety of sample matrices.

All certified standards were purchased from Wellington Laboratories (Guelph, ON, Canada), Chiron (Trondheim, Norway), or Sigma Aldrich (St. Louis, MO). The experimental workflow for the standards is shown in Fig. 1. First, the standards were diluted in methanol (Thermo Scientific; Waltham, MA) to a concentration of approximately 500 ng/mL in 1.5 mL microcentrifuge tubes (Thermo Scientific; Waltham, MA). Two hundred microliters of each diluted standard were then transferred to a 2 mL polypropylene LC autosampler vial fitted with a polypropylene insert (Agilent Technologies; Santa Clara, CA). Stock and working solutions in microcentrifuge tubes were stored at −20 °C. LC autosampler vials were stored at −20 °C until analysis (2 days) and were refilled from working solutions as necessary throughout the course of analysis (approximately 2 weeks). Optima^TM^ LC-MS grade methanol and water and ammonium acetate salt used for chromatography solvents were purchased from Fisher Scientific (Waltham, MA).

### Instrumentation

As previously described by Dodds *et al*., data was collected on a non-targeted platform which combines reversed-phase liquid chromatography, drift tube ion mobility spectrometry and mass spectrometry (LC-DTIMS-HRMS)^24^. All analyses were performed on an Agilent 1290 Infinity UPLC system (Santa Clara, CA) coupled to an Agilent 6560 IM-quadrupole time-of-flight (QTOF) platform (Santa Clara, CA) with a commercial gas kit and a MKS Instruments precision flow controller (Andover, MA). Samples were ionized using either an Agilent JetStream electrospray ionization source (ESI) and/or Agilent’s Multimode Source via atmospheric pressure chemical ionization (APCI) (Santa Clara, CA).

### Sample injection

All standards were first analyzed using flow injection analysis (FIA) wherein 10 µL of the 500 ng/mL solution was introduced to the ion source (ESI or APCI) without chromatographic separation in both negative and positive modes, with source parameters listed in Table 1. From this initial injection, it was determined which of the PFAS (Supplementary Table S1) ionized in each mode and source. Sufficient ionization was defined as appreciable signal with a mass error of less than 10 ppm for the expected ion. Mass spectra were assessed in negative mode for the [M-H]^−^, [M-H-CO_2_]^−^, or [M + Cl]^−^ ions and in positive mode for [M + H]^+^, [M + NH_4_]^+^, [M + Na]^+^, and [M + K]^+^ ions as well as their multimers. Other ion types such as [M-H-CO_2_-HF]^−^, [M-C_2_H_4_-OH]^−^, [M-CH_2_-CO_2_]^−^, [M-2H + Na]^−^, [M-2H + K]^−^, and [M + CH_3_O]^−^ were identified if high signal intensity was observed in the nested spectra.

**Table 1:** Electrospray Ionization (ESI) and Atmospheric Pressure Chemical Ionization (APCI) Source Parameters.

Parameter	ESI	APCI
Gas Temperature (°C)	230	300
Drying Gas (L/min)	11	5
Nebulizer (psi)	45	45
Sheath Gas Flow (L/min)	11	—
Sheath Gas Temperature (°C)	350	—
Capillary Voltage (V)	3500	2000
Nozzle Voltage (V)	500	—
Vaporizer (°C)	—	250
Corona (μA)	—	4

These parameters are consistent with Agilent default parameters for APCI and those described in Agilent Technologies’ application note for analysis of PFAS when using ESI^28,37,38^.

All PFAS standards with ion signals in either positive or negative mode were then run with a reversed-phase liquid chromatography gradient at least twice, resulting in two retention time (RT) replicates and triplicate CCS value analyses. Since chromatography occurs before ionization, molecules will maintain the same RT regardless of ionization source or polarity, but exact RTs are likely to vary when analyzing environmental and clinical samples due to matrix effects of the extract. Thus, these values provide a rough estimate of RT. For the chromatography analyses, 10 µL of each standard was injected onto an Agilent ZORBAX Plus C18 guard column (2.1 × 5 mm, 1.8 μm; Santa Clara, CA) followed by an Agilent ZORBAX Eclipse Plus C18 column (2.1 × 50 mm, 1.8 μm; Santa Clara, CA) with the column compartment held at 30 °C and a flow rate of 0.4 mL per minute. Mobile Phase A consisted of 100% water, while Mobile Phase B was comprised of 95% methanol and 5% water. Both mobile phases were buffered with 5 mM ammonium acetate (+/−5%). The specific LC gradient and parameters used are further described in Table 2.

**Table 2:** Reversed-Phase Liquid Chromatography (RPLC) Gradient.

Time (min)	% MPB
0.0	10
0.5	10
2.0	30
14.0	95
14.5	100
16.5	100
16.5–22.5	10

These parameters are consistent with analysis of PFAS in extracted environmental and clinical samples as adapted from an Agilent application note for PFAS analyses^37^.

### IMS and MS

IMS and HRMS measurements were collected using the Agilent 6560 IM-QTOF, using a workflow and method consistent with numerous publications^12,14,24,30^. Briefly, following ionization by either ESI or APCI in either positive or negative mode, ions are pulsed into the drift tube filled with nitrogen buffer gas (Ultra High Purity (99.999%), Airgas; Radnor, PA) held at 3.95 Torr. A trap fill time of 10000 μs and release time of 100 μs were used to increase signal intensity while minimizing peak broadening. Further IMS analyses parameters are also outlined in Table 3, and standard for using the single field method with a uniform electric field of ~17 V/cm^28^. HRMS data was collected in MS1-only mode with the time-of-flight (TOF) mass spectrometer operating in high sensitivity (2 GHz) mode for the 50–1700 *m/z* mass range.

**Table 3:** Drift Tube Ion Mobility Spectrometry Parameters.

Parameter	Value
Trap Fill	10000 μs
Trap Release	100 μs
Drift Tube Entrance (Single-Field only)^†^	1574 V
Drift Tube Exit	224 V
Rear Funnel Entrance	217.5 V
Rear Funnel Exit	45 V
Maximum Allowed Drift Time	60 ms

Voltages shown were those used for positive mode, while negative polarity mode utilized the negative values of each.

^†^Drift tube entrance voltage was used for all replicates in the single field method. For the stepped field method values for the APCI(-) tune mix ions analyses, please consult the main text.

Calibration of the instrument in both the MS and IMS dimensions is critical to obtaining reproducible CCS values. Thus, on each analysis day, a tune file was collected using FIA and the same source and instrument parameters with the Agilent ESI-L Low Concentration or Agilent APCI-Low Concentration tuning mix immediately before all subsequent data files were acquired. In the case of ESI(−/+) and APCI(+) data acquisition, these files enabled single field calibration of observed drift times^28^. In the case of APCI(−) acquisition, “gold standard” CCS values for Agilent APCI tuning mix ions do not exist and thus, the stepped field method described by Stow *et al*.^28^ was used to calculate CCS values for these ions. In the stepped field method, all IMS parameters in Table 3 were utilized except for changing the drift tube entrance voltages by 100 V at every 0.5-minute interval, beginning at 1074 V and ending at 1674 V. Using the “Multi-field” calibration option in MassHunter Acquisition software, CCS values for each calibrant ion were calculated. These values were then used in the single field calibration method to determine CCS values of ions in APCI(-).

### Data acquisition and processing

**Fig. 2:**
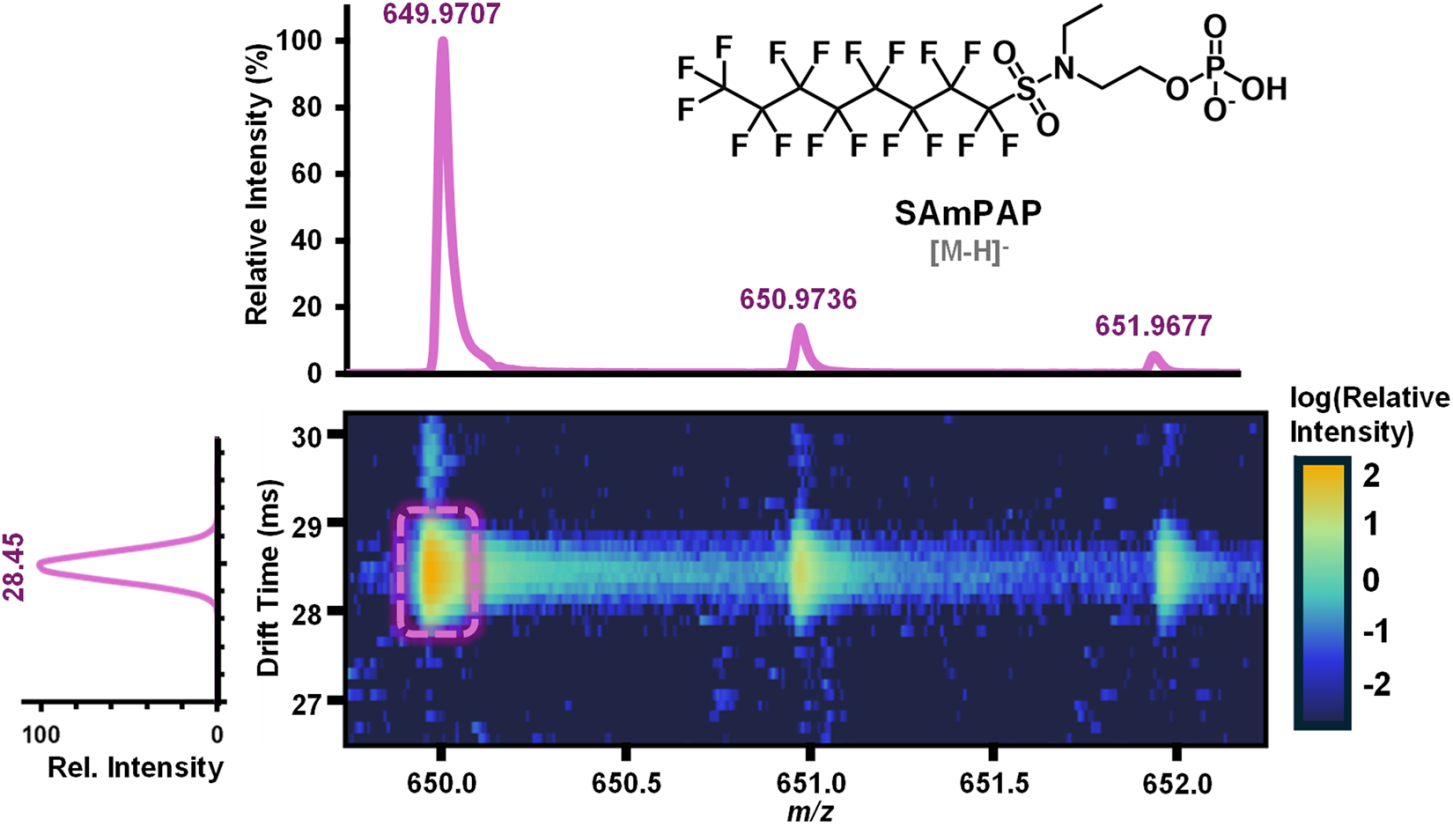
Drift time and exact mass determination from a raw data file of the PFAS SAmPAP. IM-MS Browser facilitates analysis through manual filtering and integration in the *m/z* and drift time dimensions where integration bounds are drawn around the monoisotopic mass signal to determine the corresponding drift time and *m/z* value. In the case of the [M-H]^−^ ion for SAmPAP, the software determined the *m/z* of the monoisotopic mass to be 649.9707 with a drift time of 28.45 milliseconds.

**Fig. 3:**
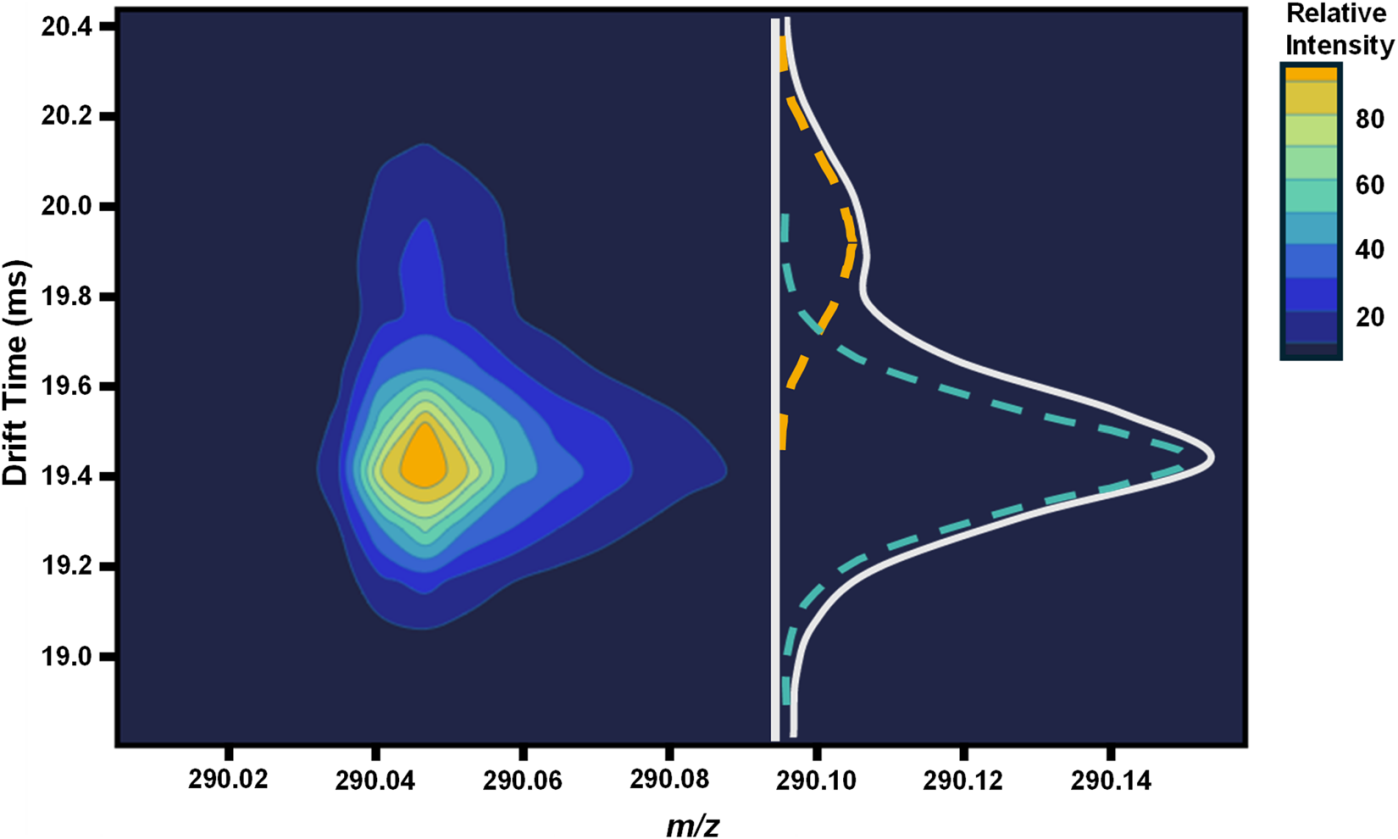
IMS-MS nested spectrum for the deprotonated form of PubChem CID 625950 or 6-(1,1,1,3,3,3-hexafluoropropan-2-yloxy)-2-*N*-methyl-1,3,5-triazine-2,4-diamine. Two different forms were observed for this molecule. The major compact form (CCS = 148.07 Å^2^) is highlighted by the teal trace and the minor extended form (CCS = 151.23 Å^2^) by the orange trace. Total abundance is represented by the white trace.

LC-IMS-HRMS data was acquired using MassHunter Acquisition Software B.09 and the resulting Agilent “.d” files were analyzed in IM-MS Browser 10.0. From each data file, the monoisotopic mass was identified, its corresponding drift time noted (Fig. 2), and these values recorded in an Excel workbook. This.xlsx workbook calculates the CCS value for each replicate based on the single field method, as well as the average CCS value and relative standard deviation. In cases where the same analyte presents two different drift times, neither of which are related to the breakdown of in-source multimers, the isomers or conformers are indicated as either “a” or “b,” representing the compact and extended forms (Fig. 3). For the dataset, two analytes are noted to have both a and b forms, 8HPFOA and a PFAS with the PubChem Compound Identifier (CID) 625950.

## Data Records

This dataset is available at Zenodo^31^ (10.5281/zenodo.14341321) as an Excel workbook and is formatted to facilitate multidimensional analyses in the Skyline software^32^. This file is also available at the Baker Lab database webpage (https://tarheels.live/bakerlab/databases/). The raw .d files for this dataset are deposited at MassIVE, a repository for mass spectrometry data, with accession number MSV000096020^33^.

For the 175 PFAS noted in the multidimensional dataset, 281 ion types were detected, including CCS values for 30 analytes reported for the first time. The ion types included [M-H]^−^, [M-H-CO_2_]^−^, [M + Cl]^−^, [M + H]^+^, [M + NH_4_]^+^, [M + Na]^+^, [M + K]^+^, [M-H-CO_2_-HF]^−^, [M-C_2_H_4_-OH]^−^, [M-CH_2_-CO_2_]^−^, [M-2H + Na]^−^, [M-2H + K]^−^, and [M + CH_3_O]^−^, as well as related multimers. This information presents an argument for analysis of PFAS in positive mode whereas many PFAS are studied only in negative mode, however for our dataset we observed 169 PFAS in negative mode, 14 in positive mode, and 8 ions in both modes. These 281 PFAS precursor CCS values (generated via ESI and/or APCI) are expected to advance analytical analyses and machine learning studies. For each newly added molecule in the dataset, the associated ions in both positive and negative modes are noted along with the RT and CCS values as well as precursor *m/z*, CAS number, PubChem CID, SMILES, full name, molecule group and vendor of the standard, which can be found in the “New Additions to PFAS Library” workbook at the same Zenodo record as previously described^31^.

## Technical Validation

**Fig. 4:**
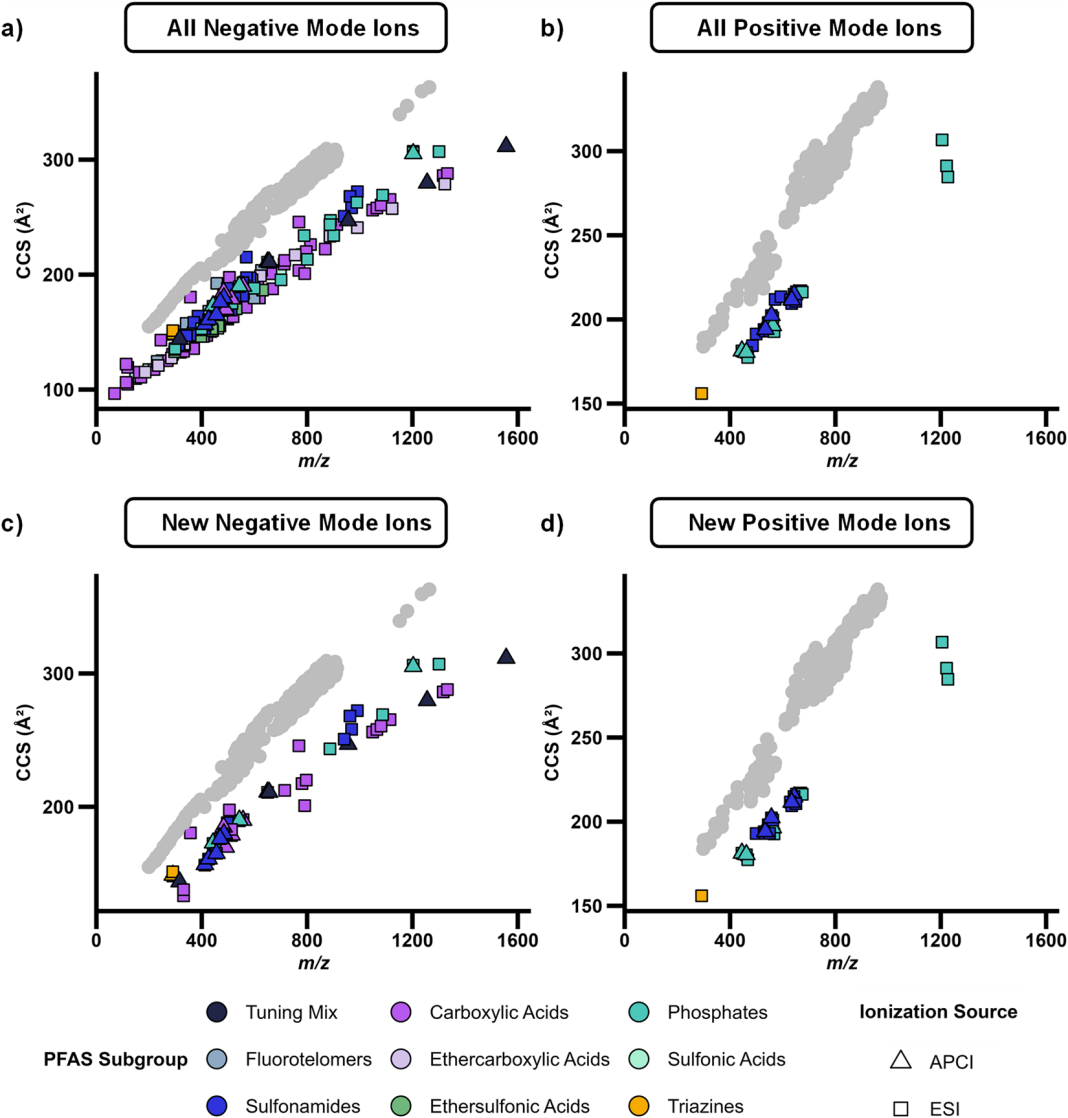
CCS versus *m/z* trendlines for characterized PFAS, excluding mass-labelled standards. Here all PFAS in the library are shown in the top row for the (**a)** negative and (**b)** positive mode ions, while the bottom row illustrated CCS values for the new PFAS standards in (**c)** negative and (**d)** positive mode. All PFAS in the dataset easily separate from the more hydrogenated molecules such as lipids which are shown as grey circles^34^. Ionization in either negative mode or positive mode or via ESI or APCI did not impact this trend.

The dataset reported here was carefully constructed with high purity standards (>92% purity reported by manufacturers). The multidimensional separations also aided in high confidence measurements. For example, highly fluorinated compounds like PFAS occupy a specific region of the CCS vs. *m/z* space when analyzed with IMS-MS. This occurs because although fluorine atoms have a similar atomic radius to hydrogen atoms and thus occupy similar volume, they are much heavier in mass (18.9984 Da versus 1.0078 Da). Thus, when evaluating a plot of CCS vs. *m/z*, PFAS fall on an isolated trendline with a lower slope relative to biological molecules as previously described by Foster *et al*.^29^, which is useful not only for evaluating PFAS in environmental matrices but also for ensuring quality and consistency of CCS calculations in this study^8,24^. As shown in Fig. 4, all PFAS CCS values calculated in this study fall along a distinct linear trendline below the biological space (shown in grey)^34^, regardless of ionization source or mode. Of further interest is the distinction between the different classes as the PFCAs which have carboxylic acid head groups are easily distinguished from the phosphates and other PFAS subclasses. Additionally, PFAS that fall closer to the biological trendline are noted as being more hydrogenated, having double bonds, or having a lower ratio of fluorine to carbon atoms, such as FDUEA and its dimer.

**Fig. 5:**
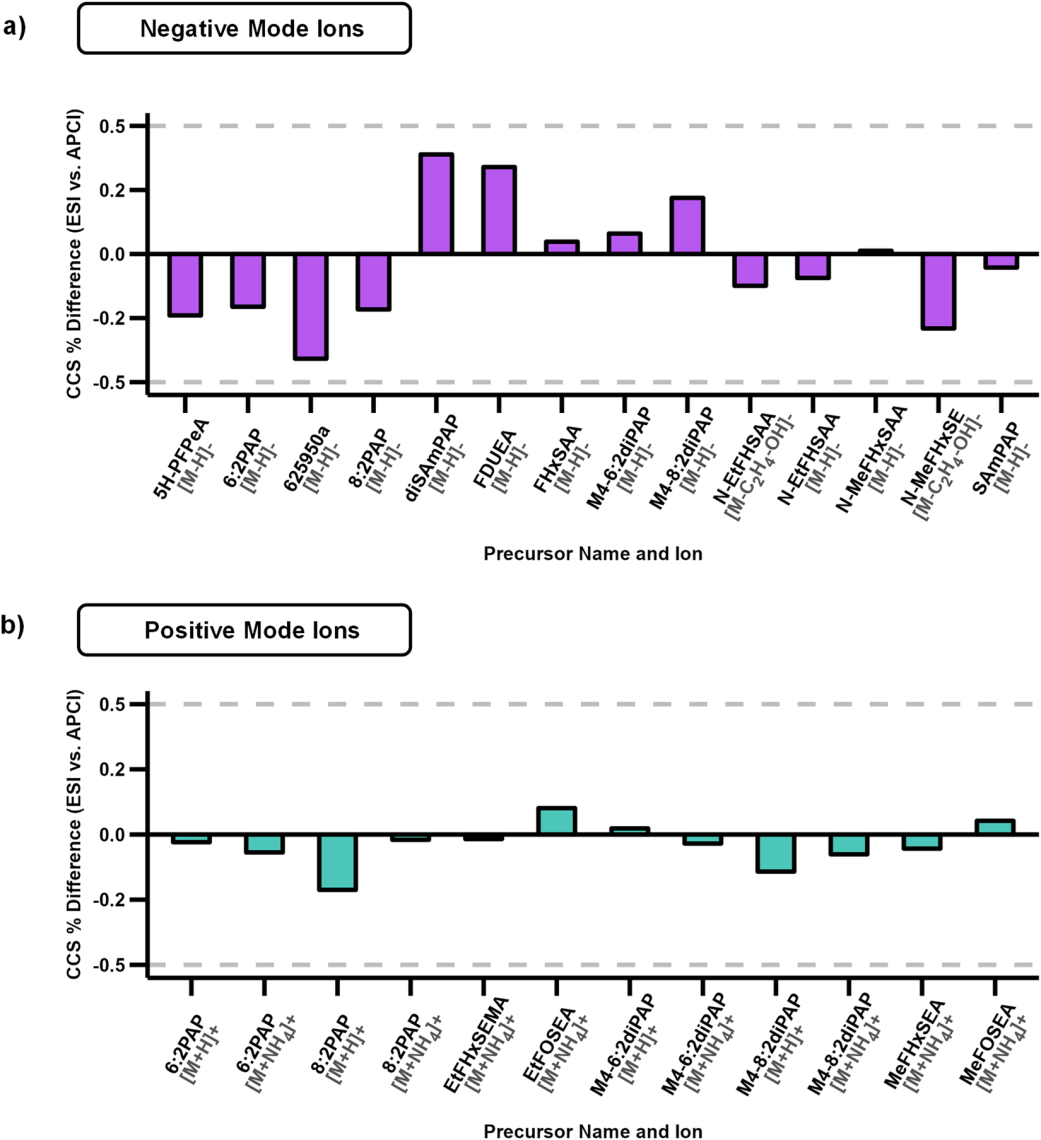
Percent difference between ESI and APCI derived ion CCS values. The percent difference between precursor ions formed with both ionization sources was less than 0.5% for all occurrences in both (**a)** negative mode and (**b)** positive mode.

In the MS analyses, all identified ions had a mass error of less than 10 ppm across all replicates, which is within the expected error of this instrument. Additionally, all CCS values calculated using the single field method across triplicate injections had a relative standard deviation of less than 0.3%, which is also within the expected range of error for this platform. In the cases in which molecules ionized to form the same adduct via both ESI and APCI, the percent difference in CCS values were less than 0.5% in negative mode and less than 0.25% in positive mode (Fig. 5).

**Fig. 6:**
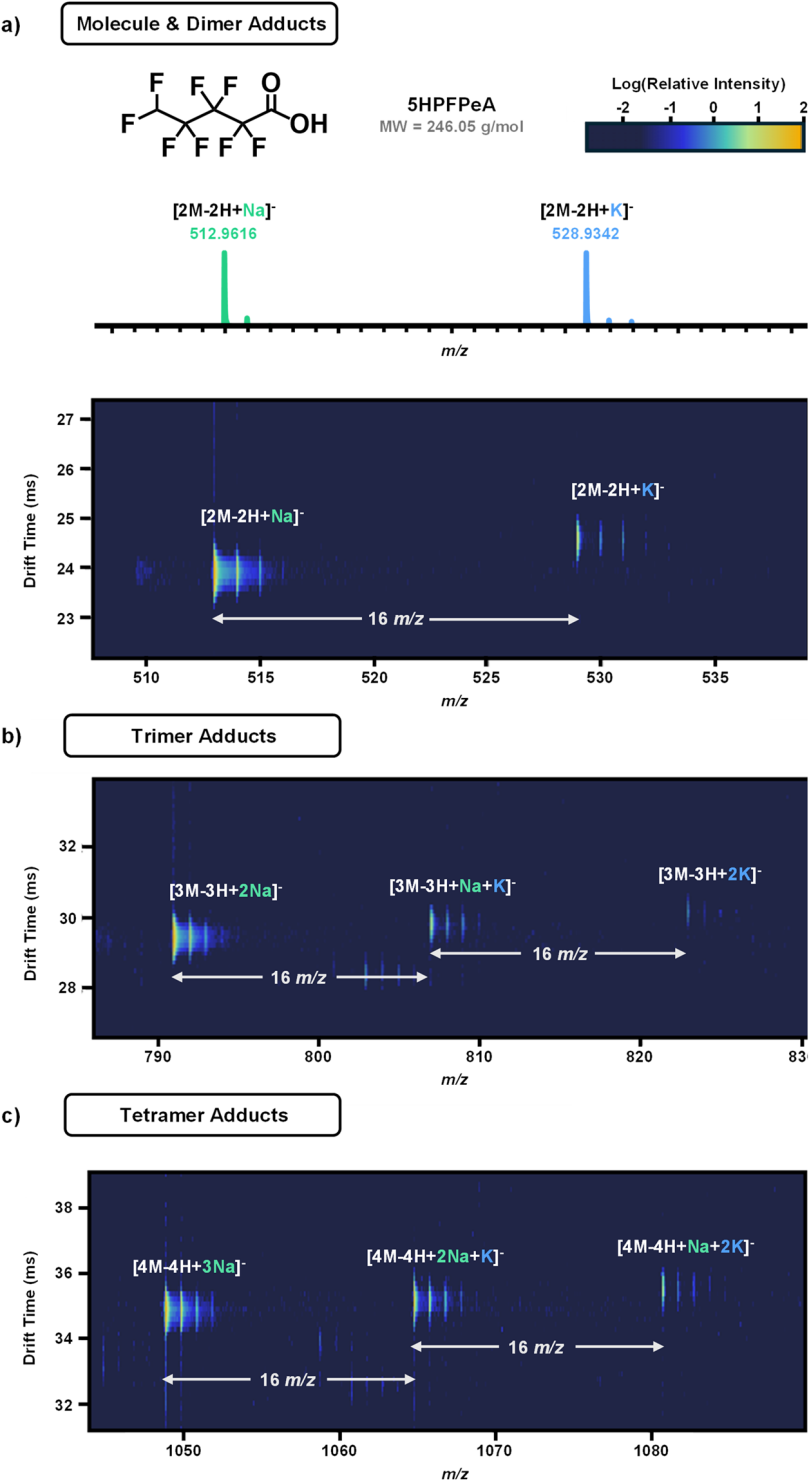
5H-Octafluoropentanoic acid (5H-PFPeA) multimers with sodiated and potassiated adduct pattern. (**a)** Structure of 5H-PFPeA and molecular weight. Sodiated and potassiated adducts show expected isotopic distribution and spacing in the *m/z* dimension and in the IMS-MS nested spectra of the dimer. (**b)** The trimer and (**c)** tetramer of 5H-PFPeA also show the same pattern of alkali metal adductions, where drift time increases as Na^+^ are replaced by K^+^ as adduct ions.

Assessment of the different ion types with LC-DTIMS-HRMS also showed the formation of multimers for 15 PFAS analytes in negative mode with some forming up to pentamers. In some cases, the patterns in alkali metal adducts were used to increase confidence that observed signals are correctly assigned to their adduct formulas (Fig. 6). For example, sodium and potassium adducted to 5H-PFPeA allowing assignment of its dimer, trimer, and tetramer.

Evaluation of the PFAS standards via APCI was also necessary as three ions were only observed in APCI and not ESI. However, there was a challenge with these assessments as there are no CCS values currently noted for the APCI calibrants in negative mode as the Agilent APCI-Low Concentration tune mix ion CCS values are not validated by an interlaboratory study. However, DTIMS provides the ability to calculate CCS values from first principles with the stepped field method, so these measurements were conducted here to provide values for those coupling an APCI source with any IMS platform capable of calculating CCS values. To validate these results, stepped field data was collected for both the ESI and APCI sources in negative mode and the sources were alternated to make sure error was consistent throughout the three replicates. In comparing ESI-Low tune mix ion CCS values calculated via the stepped field method to their “gold standard” value validated in the interlaboratory study by Stow *et al*.^28^, the percent error for all ions was less than 0.5% (Table 4). Therefore, it is expected that the APCI values also noted in Table 4 should have similar errors. To reduce errors further, users should conduct the stepped field experiment in their own laboratory to determine the Agilent APCI-L tuning mix ion CCS values, since the effective length of the drift tube may vary slightly between instruments.

**Table 4:** Validation of Stepped Field Method Applied to APCI Tune Mix Ions.

*m/z*	Mode	Average Experimental CCS (Å^2^) (n = 3)	Theoretical^1^ CCS (Å^2^)	Error between Theoretical and Experimental (%)
301.9981	**ESI(-)**	139.8	140.0	**0.20**
601.9798	**ESI(-)**	181.0	180.8	**−0.11**
1033.9881	**ESI(-)**	255.4	255.3	**−0.04**
1333.9689	**ESI(-)**	285.7	284.8	**−0.34**
1633.9498	**ESI(-)**	318.7	319.0	**0.11**
316.0138	**APCI(-)**	143.7	—	—
655.9911	**APCI(-)**	210.9	—	—
955.9719	**APCI(-)**	246.9	—	—
1255.9528	**APCI(-)**	279.7	—	—
1555.9336	**APCI(-)**	311.5	—	—

Theoretical values for the ESI tune mix ions were used from Ref. ^28^.

CCS values reported in this dataset are not significantly impacted by changes to the LC methods presented in this paper. Although the use of different LC solvents may impact RT and elution order, solvent composition is known to account for less than 0.5% difference in CCS values, which is within the expected range of IMS analysis platforms and thus these values are still suitable for database matching^35^.

## Usage Notes

This work provides access to LC, DTIMS, and MS characteristics for 175 PFAS and 281 PFAS ions including [M-H]^−^, [M-H-CO_2_]^−^, [M + Cl]^−^, [M + H]^+^, [M + NH_4_]^+^, [M + Na]^+^, [M + K]^+^, [M-H-CO_2_-HF]^−^, [M-C_2_H_4_-OH]^−^, [M-CH_2_-CO_2_]^−^, [M-2H + Na]^−^, [M-2H + K]^−^, and [M + CH_3_O]^−^, as well as related multimers. The detailed multidimensional RPLC-DTIMS-HRMS dataset contains RPLC retention times, *m/z* values for precursor, and CCS values for each substance. It also provides 30 additional PFAS CCS values based on analytical standards which have not yet been included in any publications. While this dataset was built using a specific RPLC gradient, and platform with DTIMS, these values could pertain to other RPLC gradient lengths and IMS instruments. Each separation dimension can also be ignored if not required by the researcher. This RPLC-DTIMS-HRMS dataset resource is available on Zenodo^31^ and at the Baker Lab website, both of which will be updated regularly as more identifications are made and new chemical standards are studied.

CCS values derived from this study were uploaded to PubChem using FAIR (Findable, Accessible, Reproducible, Interoperable) templates available on PubChem^36^ (filled templates are available on GitLab, https://gitlab.com/uniluxembourg/lcsb/eci/pubchem/-/tree/master/annotations/CCS/BakerLab). The corresponding data can be downloaded from the BakerLab Data Source page in PubChem (https://pubchem.ncbi.nlm.nih.gov/source/25763) and browsed via the classification tree developed from this dataset and another lipid dataset (https://pubchem.ncbi.nlm.nih.gov/classification/#hid=124). They are also available via other PubChem interfaces (as described in the PubChem documentation and via code snippets provided on GitLab - https://gitlab.com/uniluxembourg/lcsb/eci/pubchem).

To use the multidimensional PFAS dataset for targeted analyses of environmental and clinical samples in the software Skyline^32^, copy and paste columns B-G of the “Skyline Formatted Library” directly into the transition list. A more detailed description of how to create a Skyline document and use this software for targeted analyses can be found in Ref. ^33^. It is also recommended that users select the appropriate sheet within the workbook to reflect their data collection method(s).

## Supplementary information

Supplementary Table S1

## Data Availability

In this study, RStudio was used for data visualization and figure creation. Microsoft Excel was used for calculating CCS values from drift times, as well as statistical analyses of relative standard deviation and mass error. An example fillable Excel workbook of the “Single Field Template” used to calculate the CCS values from extracted drift times is available as part of the Zenodo record^31^. Code and data related to the CCS integration in PubChem is available on GitLab (https://gitlab.com/uniluxembourg/lcsb/eci/pubchem), which also includes example scripts to retrieve the CCS data from PubChem in R.

## References

[1] Gaines, L. G. T. Historical and current usage of per‐ and polyfluoroalkyl substances (PFAS): A literature review. *American Journal of Industrial Medicine* **66**, 353–378, 10.1002/ajim.23362 (2023). 35614869 10.1002/ajim.23362

[2] Schymanski, E. L. *et al*. Per- and Polyfluoroalkyl Substances (PFAS) in PubChem: 7 Million and Growing. *Environmental Science & Technology* **57**, 16918–16928, 10.1021/acs.est.3c04855 (2023). 37871188 10.1021/acs.est.3c04855 PMC10634333

[3] Wang, Z. *et al*. A New OECD Definition for Per- and Polyfluoroalkyl Substances. *Environmental Science & Technology* **55**, 15575–15578, 10.1021/acs.est.1c06896 (2021). 34751569 10.1021/acs.est.1c06896

[4] Hammel, E., Webster, T. F., Gurney, R. & Heiger-Bernays, W. Implications of PFAS definitions using fluorinated pharmaceuticals. *iScience* **25**, 104020, 10.1016/j.isci.2022.104020 (2022). 35313699 10.1016/j.isci.2022.104020 PMC8933701

[5] Brase, R. A., Mullin, E. J. & Spink, D. C. Legacy and Emerging Per- and Polyfluoroalkyl Substances: Analytical Techniques, Environmental Fate, and Health Effects. *International Journal of Molecular Sciences* **22**, 995, 10.3390/ijms22030995 (2021). 33498193 10.3390/ijms22030995 PMC7863963

[6] Schaider, L. A. *et al*. Fluorinated Compounds in U.S. Fast Food Packaging. *Environmental Science & Technology Letters* **4**, 105–111, 10.1021/acs.estlett.6b00435 (2017). 30148183 10.1021/acs.estlett.6b00435 PMC6104644

[7] Whitehead, H. D. *et al*. Fluorinated Compounds in North American Cosmetics. *Environmental Science & Technology Letters* **8**, 538–544, 10.1021/acs.estlett.1c00240 (2021).

[8] Luo, Y.-S. *et al*. Rapid Characterization of Emerging Per- and Polyfluoroalkyl Substances in Aqueous Film-Forming Foams Using Ion Mobility Spectrometry–Mass Spectrometry. *Environmental Science & Technology* **54**, 15024–15034, 10.1021/acs.est.0c04798 (2020). 33176098 10.1021/acs.est.0c04798 PMC7719402

[9] Brusseau, M. L., Anderson, R. H. & Guo, B. PFAS concentrations in soils: Background levels versus contaminated sites. *Science of The Total Environment* **740**, 140017, 10.1016/j.scitotenv.2020.140017 (2020). 32927568 10.1016/j.scitotenv.2020.140017 PMC7654437

[10] Hall, S. M. *et al*. PFAS levels in paired drinking water and serum samples collected from an exposed community in Central North Carolina. *Science of The Total Environment* **895**, 165091, 10.1016/j.scitotenv.2023.165091 (2023). 37355130 10.1016/j.scitotenv.2023.165091 PMC10529814

[11] Smalling, K. L. *et al*. Per- and polyfluoroalkyl substances (PFAS) in United States tapwater: Comparison of underserved private-well and public-supply exposures and associated health implications. *Environment International* **178**, 108033, 10.1016/j.envint.2023.108033 (2023). 37356308 10.1016/j.envint.2023.108033

[12] Dodds, J. N. *et al*. Evaluating Solid Phase Adsorption Toxin Tracking (SPATT) for passive monitoring of per- and polyfluoroalkyl substances (PFAS) with Ion Mobility Spectrometry-Mass Spectrometry (IMS-MS. *Science of The Total Environment* **947**, 174574, 10.1016/j.scitotenv.2024.174574 (2024). 38981548 10.1016/j.scitotenv.2024.174574 PMC11295640

[13] McCord, J. & Strynar, M. Identification of Per- and Polyfluoroalkyl Substances in the Cape Fear River by High Resolution Mass Spectrometry and Nontargeted Screening. *Environmental Science & Technology* **53**, 4717–4727, 10.1021/acs.est.8b06017 (2019). 30993978 10.1021/acs.est.8b06017 PMC7478245

[14] Kirkwood, K. I. *et al*. Utilizing Pine Needles to Temporally and Spatially Profile Per- and Polyfluoroalkyl Substances (PFAS. *Environmental Science & Technology* **56**, 3441–3451, 10.1021/acs.est.1c06483 (2022). 35175744 10.1021/acs.est.1c06483 PMC9199521

[15] Worley, R. R. *et al*. Per- and polyfluoroalkyl substances in human serum and urine samples from a residentially exposed community. *Environment International* **106**, 135–143, 10.1016/j.envint.2017.06.007 (2017). 28645013 10.1016/j.envint.2017.06.007 PMC5673082

[16] Zheng, G. *et al*. Per- and Polyfluoroalkyl Substances (PFAS) in Breast Milk: Concerning Trends for Current-Use PFAS. *Environmental Science & Technology* **55**, 7510–7520, 10.1021/acs.est.0c06978 (2021). 33982557 10.1021/acs.est.0c06978

[17] Von Holst, H. *et al*. Perfluoroalkyl substances exposure and immunity, allergic response, infection, and asthma in children: review of epidemiologic studies. *Heliyon* **7**, e08160, 10.1016/j.heliyon.2021.e08160 (2021). 34712855 10.1016/j.heliyon.2021.e08160 PMC8529509

[18] Fletcher, T. *et al*. Associations between PFOA, PFOS and changes in the expression of genes involved in cholesterol metabolism in humans. *Environment International* **57-58**, 2–10, 10.1016/j.envint.2013.03.008 (2013). 23624243 10.1016/j.envint.2013.03.008

[19] Blake, B. E. & Fenton, S. E. Early life exposure to per- and polyfluoroalkyl substances (PFAS) and latent health outcomes: A review including the placenta as a target tissue and possible driver of peri- and postnatal effects. *Toxicology* **443**, 152565, 10.1016/j.tox.2020.152565 (2020). 32861749 10.1016/j.tox.2020.152565 PMC7530144

[20] Winquist, A. *et al*. Case–Cohort Study of the Association between PFAS and Selected Cancers among Participants in the American Cancer Society’s Cancer Prevention Study II LifeLink Cohort. *Environmental Health Perspectives* **131**, 10.1289/ehp13174 (2023). 10.1289/EHP13174 PMC10718084 38088576

[21] Hale, S. E., Arp, H. P. H., Schliebner, I. & Neumann, M. What’s in a Name: Persistent, Mobile, and Toxic (PMT) and Very Persistent and Very Mobile (vPvM) Substances. *Environmental Science & Technology* **54**, 14790–14792, 10.1021/acs.est.0c05257 (2020). 33170664 10.1021/acs.est.0c05257

[22] Phillis, M. Biden administration sets first-ever limits on ‘forever chemicals’ in drinking water. *Associated Press* (2024).

[23] PFAS Team. *PFAS Technical and Regulatory Guidance Document and Fact Sheets*, 2023).

[24] Dodds, J. N., Hopkins, Z. R., Knappe, D. R. U. & Baker, E. S. Rapid Characterization of Per- and Polyfluoroalkyl Substances (PFAS) by Ion Mobility Spectrometry–Mass Spectrometry (IMS-MS). *Analytical Chemistry* **92**, 4427–4435, 10.1021/acs.analchem.9b05364 (2020). 32011866 10.1021/acs.analchem.9b05364 PMC7173758

[25] Joerss, H. & Menger, F. The complex ‘PFAS world’ - How recent discoveries and novel screening tools reinforce existing concerns. *Current Opinion in Green and Sustainable Chemistry* **40**, 100775, 10.1016/j.cogsc.2023.100775 (2023).

[26] Valdiviezo, A. *et al*. Analysis of per- and polyfluoroalkyl substances in Houston Ship Channel and Galveston Bay following a large-scale industrial fire using ion-mobility-spectrometry-mass spectrometry. *Journal of Environmental Sciences* **115**, 350–362, 10.1016/j.jes.2021.08.004 (2022). 10.1016/j.jes.2021.08.004 PMC8724578 34969462

[27] Dodds, J. N. & Baker, E. S. Ion Mobility Spectrometry: Fundamental Concepts, Instrumentation, Applications, and the Road Ahead. *Journal of the American Society for Mass Spectrometry* **30**, 2185–2195, 10.1007/s13361-019-02288-2 (2019). 31493234 10.1007/s13361-019-02288-2 PMC6832852

[28] Stow, S. M. *et al*. An Interlaboratory Evaluation of Drift Tube Ion Mobility–Mass Spectrometry Collision Cross Section Measurements. *Analytical Chemistry* **89**, 9048–9055, 10.1021/acs.analchem.7b01729 (2017). 28763190 10.1021/acs.analchem.7b01729 PMC5744684

[29] Foster, M. *et al*. Uncovering PFAS and Other Xenobiotics in the Dark Metabolome Using Ion Mobility Spectrometry, Mass Defect Analysis, and Machine Learning. *Environmental Science & Technology* **56**, 9133–9143, 10.1021/acs.est.2c00201 (2022). 35653285 10.1021/acs.est.2c00201 PMC9474714

[30] Kirkwood-Donelson, K. I., Dodds, J. N., Schnetzer, A., Hall, N. & Baker, E. S. Uncovering per- and polyfluoroalkyl substances (PFAS) with nontargeted ion mobility spectrometry–mass spectrometry analyses. *Science Advances* **9**, eadj7048, 10.1126/sciadv.adj7048. 10.1126/sciadv.adj7048 PMC10599621 37878714

[31] Joseph, K. *et al*. Dataset for “Multidimensional library for the improved identification of per-and polyfluoroalkyl substances (PFAS)”. Zenodo 10.5281/zenodo.14341321 (2024). 10.1038/s41597-024-04363-0 PMC11763048 39863618

[32] MacLean, B. *et al*. Skyline: an open source document editor for creating and analyzing targeted proteomics experiments. *Bioinformatics* **26**, 966–968, 10.1093/bioinformatics/btq054 (2010). 20147306 10.1093/bioinformatics/btq054 PMC2844992

[33] Joseph, K. *et al*. Data for Multidimensional Library for the Improved Identification of Per- and Polyfluoroalkyl Substances (PFAS). MassIVE 10.25345/C5XW4876Q (2024). 10.1038/s41597-024-04363-0 PMC11763048 39863618

[34] Solosky, A. M., Kirkwood-Donelson, K. I., Odenkirk, M. T. & Baker, E. S. Recent additions and access to a multidimensional lipidomic database containing liquid chromatography, ion mobility spectrometry, and tandem mass spectrometry information. *Analytical and Bioanalytical Chemistry*10.1007/s00216-024-05351-4 (2024). 10.1007/s00216-024-05351-4 PMC11427178 38814344

[35] Chung, N. A., May, J. C., Robinson, R. A. S. & McLean, J. A. Solvent Composition Can Have a Measurable Influence on the Ion Mobility-Derived Collision Cross Section of Small Molecules. *Journal of the American Society for Mass Spectrometry* **35**, 234–243, 10.1021/jasms.3c00338 (2024). 38082535 10.1021/jasms.3c00338

[36] Schymanski, E. L. & Bolton, E. E. FAIR chemical structures in the Journal of Cheminformatics. *Journal of Cheminformatics* **13**10.1186/s13321-021-00520-4 (2021). 10.1186/s13321-021-00520-4 PMC8262078 34229711

[37] Anumol, T., Yang, D.-H. D., Sosienski, T. & Batoon, P. Analysis of per/polyfluoroalkyl substances (PFASs) in drinking water using the Agilent Ultivo triple quadrupole LC/MS. *Agilent Technologies, Inc*. (2018).

[38] Fisher, S. & Duncan, W. Optimizing the Agilent Multimode Source. *Agilent Technologies, Inc*. (2007).

