# Supplementary material for: Multidimensional library for the improved identification of per- and polyfluoroalkyl substances (PFAS)
Source: Sci Data. 2025 Jan 25;12:150. doi: 10.1038/s41597-024-04363-0 (PMC11763048; doi:10.1038/s41597-024-04363-0)
Supplement: Supplementary file 1 — Supplementary Table S1 [file 41597_2024_4363_MOESM1_ESM.pdf]

Supplementary Table S1. Summary of analyzed standards and ionization success for each source/mode.

Molecule	PubChem CID	ESI(-)	APCI (-)	ESI(+)	APCI(+)
6:2PAP	165362396	Y	Y	Y	Y
8:2PAP	138394457	Y	Y	Y	Y
8:2FTAcr	119747	N	N	N	N
10:2FTAcr	87274	N	N	N	N
8:2FTOAc	12933490	N	N	N	N
10:2FTOAc	138395138	N	N	N	N
diSAmPAP	138395141	Y	Y	Y	N
FDUEA	101674537	Y	Y	N	N
FHUEA	85976247	Y	N	N	N
M4-6:2diPAP	138394469	Y	Y	Y	Y
M4-8:2diPAP	138394471	Y	Y	Y	Y
MFDEA	165360305	Y	N	N	N
MFDUEA	165360307	Y	Y	N	N
MFHEA	138394397	Y	N	N	N
MFHUEA	165360306	Y	N	N	N
MFOEA	165360304	Y	N	N	N
MFOUEA	165360297	Y	N	N	N
SAmPAP	133082391	Y	Y	Y	N
625950	625950	Y	Y	Y	High CCS RSD and mass error
1H-Perfluoroheptane	9778	Same adduct as PFOA	N	N	N
44480566	44480566	N	N	N	N
2778357	2778357	N	N	N	High CCS RSD and mass error
2776755	2776755	N	N	N	N
5H-PFPeA	120227	Y	Y	N	N
550340	550340	N	N	N	N
108404	108404	N	N	N	N
533990	533990	N	N	N	N
2769669	2769669	N	N	N	High CCS RSD and mass error
3:3 FTCA	2774909	Y	Y	N	N
21360390	21360390	N	N	N	N
3:2FTOH	2782534	N	N	N	N
12017056	12017056	N	N	N	N
6:3FTOH	2776181	N	N	N	N
7Me-6:1FTOH	2776232	N	N	N	N
8:2FTMAC	160606	N	N	N	N
8HPFOA	14922999	Y	Y	N	N

Molecule	PubChem CID	ESI(-)	APCI (-)	ESI(+)	APCI(+)
EtFHxSEMA	106119	N	N	Y	Y
EtFOSEA	67921	N	N	Y	Y
FHxSAA	139596791	Y	Y	N	n
MeFHxSEA	105449	N	N	Y	Y
MeFOSEA	91369	N	N	Y	Y
N-EtFHSAA	105445	Y	Y	Y	N
N-MeFHxSAA	87481295	Y	N	N	N
N-MeFHxSE	110556	Y	Y	N	N
ATM2	9715	-	Y	-	-
ATM3	44717242	-	Y	-	-
ATM4	2775076	-	Y	-	-
ATM5	51342023	-	Y	-	-
ATM6	2775070	-	Y	-	-

PFAS standards analyzed with their corresponding PubChem Compound Identifiers (CIDs) and indicators of ionization detected in each source/mode at a concentration of 500 ng/mL. “Y” indicates that the molecule ionized in that source/mode and the calculated CCS value is available as of the publication date of this manuscript. “N” indicates that there were no ions detected as described in the main text. Cells with additional text indicate possible ionization and summarize why these CCS values are not included in the data repository. “-” indicates molecules with existing CCS values. It is possible that ions may be seen in these conditions at different concentrations. All standards without common abbreviations are listed as their PubChem CID.
